# Advances in the prevention, management, and treatment of community-acquired pneumonia

**DOI:** 10.12688/f1000research.7657.1

**Published:** 2016-03-08

**Authors:** Mathias W. Pletz, Gernot G. Rohde, Tobias Welte, Martin Kolditz, Sebastian Ott

**Affiliations:** 1 Center for Infectious Diseases and Infection Control, Jena University Hospital, Jena, Germany; 2 Department of Respiratory Medicine, Hannover Medical School, Hannover, Germany; 3 Department of Respiratory Medicine, Maastricht University Medical Center, Maastricht, Netherlands; 4 Division of Pulmonology, University Hospital Carl Gustav Carus, Dresden, Germany; 5 Department of Pulmonary Medicine, Inselspital, University Hospital, Bern, Switzerland

**Keywords:** community-acquired pneumonia, Streptococcus pneumoniae, pulmonary infection, CAP

## Abstract

Community-acquired pneumonia (CAP) is the infectious disease with the highest number of deaths worldwide. Nevertheless, its importance is often underestimated. Large cohorts of patients with CAP have been established worldwide and improved our knowledge about CAP by far. Therefore, current guidelines are much more evidence-based than ever before. This article discusses recent major studies and concepts on CAP such as the role of biomarkers, appropriate risk stratification to identify patients in need of hospitalisation or intensive care, appropriate empiric antibiotic therapy (including the impact of macrolide combination therapy and antibiotic stewardship), and CAP prevention with novel influenza and pneumococcal vaccines.

## Introduction and epidemiology

Community-acquired pneumonia (CAP) remains a burden in the modern world. The annual incidence ranges from between 2.7 and 10 per 1000 persons and has not changed much during the last few decades ^1^. In Germany, around 250,000 patients are hospitalised because of CAP each year, and it is expected that twice as many patients with CAP are managed in primary care ^2^. The incidence shows a U-shaped distribution from very young to very old age ^3^. CAP is still one of the most important reasons for premature death, particularly in developing countries and in children ^4^.

CAP is an infectious disease of the lung parenchyma and adjacent organs. Respiratory bacteria constitute the major group of causative organisms. However, there must be some caution, as in most studies in more than 50% of the cases no pathogen could be identified ^5^. *Streptococcus pneumoniae* is the pathogen most frequently identified throughout all studies and settings (outpatients, inpatients, and intensive care unit [ICU] patients with CAP) ^5– 7^. *Haemophilus influenzae* (HI) is also frequently detected in outpatients (13%) but much less in hospitalised patients (6% to 7%) ^5^. A possible reason is the high prevalence of HI in patients with chronic obstructive pulmonary disease (COPD) ^8^, patients who are at high risk of developing CAP ^9^. Another important group of pathogens is respiratory viruses, particularly in outpatients but also inpatients, much less in ICU patients ^5^. New detection techniques such as multiplex-polymerase chain reaction allow better insight into the relevant spectrum of viruses involved ^10^. This fast-track diagnostic also allows us to put pandemic virus emergence into perspective, as shown during the H1N1 pandemic, in which respiratory syncytial virus and human metapneumovirus were the most prevalent viruses and only pandemic influenza virus A/H1N1 (2009), and not seasonal influenza virus, was detected ^11^. Outside pandemics, seasonal influenza viruses cause yearly increases in CAP incidence and lead to increased mortality in patients co-infected with bacterial pathogens ^12, 13^. Another relevant group are the so-called “atypical” bacteria. *Mycoplasma pneumonia* is frequent in young patients with CAP (7% to 12%) and usually shows a benign course ^14, 15^. *Chlamydophila pneumoniae* historically has been reported to be a frequent pathogen mainly on the basis of serological assays. However, more recent research using molecular techniques found significantly lower detections rates (21% versus 3%, respectively) ^15^. *Legionella pneumophila* has been identified as a causative pathogen with different frequencies ^5, 16, 17^. It also occurs in outpatients, who commonly show a more favorable course of disease than inpatients ^16^.

## Biomarkers

Historically, pro-inflammatory biomarkers such as leucocyte count and C-reactive protein (CRP) are widely used in CAP. In most patients with CAP, these markers are elevated and show the highest levels in bacterial CAP, followed by atypical CAP and viral CAP ^18^. In mixed (bacterial + viral) CAP, CRP levels seem to be highest but the predictive value is low ^19^. However, an individual prediction of CAP aetiology is not possible ^18^. Also, procalcitonin (PCT), which shows a very fast response during infections, is not able to predict aetiology of CAP ^18^. However, PCT levels on admission can support the identification of severe outcomes of CAP and add to the prognostic properties of clinical risk score ^20^. It has a higher prognostic accuracy compared with CRP and leucocyte count ^21^. This has been recently confirmed ^22^. Interestingly, PCT levels can provide independent identification of patients at low risk of death within CRB-65 (confusion of new onset, respiratory rate of at least 30 breaths per minute, blood pressure of less than 90 mmHg systolic or diastolic blood pressure of 60 mmHg or less, and age of at least 65 years) classes ^21^. Importantly, antibiotic pre-treatment has to be taken into account, as it influences the prognostic properties ^23^.

A very recent study showed that the diagnostic accuracies of CRP and PCT are insufficient to confirm CAP if the diagnosis is established by using a gold standard that includes thoracic computed tomography (CT) scan ^24^. However, in primary care, the addition of CRP (optimal cutoff of more than 30 mg/L) improved the diagnosis of CAP in patients with typical signs and symptoms, whereas PCT did not add clinically relevant information ^25^.

In the most comprehensive study on the prognostic properties of new CAP biomarkers (including mid-regional pro-adrenomedullin [MR-proADM], mid-regional pro-atrial natriuretic peptide [MR-proANP], pro-arginine-vasopressin [copeptin], proendothelin-1 [CT-proET-1], PCT, CRP, white blood cell count, and CRB-65 score), MR-proADM, a cardiovascular biomarker, showed the best individual and a combination of CRB-65 with MR-proADM showed the best overall prognostic performance ^26, 27^.

Hyponatremia is common on admission among patients with CAP and was independently associated with mortality. The combination of sodium and pro-vasopressin and pro-ANP levels achieved the highest prediction of mortality in a recent analysis ^28^.

A very simple but powerful biomarker is admission blood glucose (Glc). Already mildly elevated Glc levels were significantly associated with an increased short-term mortality odds ratio (OR) of 1.56 with further increases at higher Glc levels. Therefore, acute hyperglycaemia may identify patients in need of intensified care to reduce the risk of death from CAP ^29^. Potentially along comparable mechanisms and independently of clinical scores and inflammatory biomarkers, increased serum cortisol levels have been associated with mortality in CAP ^30^. On the other hand, admission hypoglycaemia has also been associated with increased short- and long-term mortality ^31^, underlining the value of Glc as a useful biomarker.

## Risk stratification

### Prognosis

CAP shows a highly variable disease course; published mortality rates vary between less than 1% and more than 40% according to treatment setting, disease severity, age, and comorbidities ^5^. Whereas mortality and complication rates remain low in non-severe CAP managed in the community ^32^, hospitalised CAP is associated with a high risk of respiratory failure or sepsis-related organ dysfunction. CAP mortality of hospitalised patients in Germany continues to be about 13%, rising to more than 35% in patients with need of mechanical ventilation ^2^. Although treatment restrictions and functional status of multi-morbid patients might influence such mortality figures ^33^, even after conservative estimates excluding all patients residing in nursing homes or being bedridden before the CAP event, mortality remains more than 7%, which matches mortality rates of other recognised medical emergency conditions such as ST-elevation myocardial infarction ^34^. This tremendous prognostic spectrum requires timely and adequate risk stratification as a crucial first step in CAP management to determine level of care and treatment intensity. From a clinical perspective, risk stratification is divided into two major components: (1) identification of patients with a low complication risk in the outpatient setting suitable for ambulatory treatment and (2) identification of patients with high risk for acute organ failure necessitating early intensified management and monitoring interventions in the emergency department.

### Predicting low risk of complications in the outpatient setting

To assist clinical risk assessment, different score systems have been recommended by guidelines. Most established are the pneumonia severity index (PSI) score ^35^, consisting of 20 clinical, laboratory, and radiographic variables, and the CRB-65 or CURB criteria, which include only four parameters ^32, 36^. They proved to be comparable tools for mortality prediction ^37, 38^, but the CRB-65 score is preferred in the outpatient setting, as it is easy to calculate and works without laboratory parameters. However, its sensitivity in elderly and multi-morbid patients is suboptimal, and poor functional status has been shown as a major independent mortality predictor for these patients in a recent analysis ^33^. Additionally, pre-existing chronic comorbidities have been independently associated with adverse prognosis in CAP ^2, 35, 39– 42^. In particular, acute cardiac complications are of major prognostic impact ^41, 42^. Therefore, the absence of (potentially) decompensating comorbidities is another precondition for ambulatory treatment. Recent studies identified poor oxygenation as an independent predictor for complications and mortality despite a low CRB-65 score ^43– 45^. Accordingly, a score adding poor oxygenation (saturation of less than 90%) and potentially decompensating comorbidities to the CRB-65 criteria recently has been introduced and validated in a large study from the CAPNETZ cohort, showing superior low-risk prediction compared with the original criteria ^39, 40, 46^. The resulting risk evaluation to identify patients suitable for ambulatory treatment in the outpatient setting is depicted in Table 1.

**Table 1.** Low-risk identification in the outpatient setting.

Clinical assessment, supplemented by evaluation of the following criteria:
• Respiratory rate of less than 30 per min
• Diastolic/systolic blood pressure of at least 61 mm Hg/90 mm Hg
• No new-onset mental confusion
• Oxygen saturation of at least 90% on room air
• No (potentially) decompensating comorbidity
• No poor functional status (“chronically bedridden”)

### Identifying high-risk patients in need of intensified management in the emergency department

Acute pulmonary or extra-pulmonary organ dysfunction due to sepsis or decompensating (especially cardiovascular) comorbidities determines early prognosis in CAP ^41, 47– 49^. Organ dysfunction risk is highest within the first 3 days after hospitalisation ^2, 41, 48, 50, 51^. Efforts have been made to characterise a subgroup with an “emergency presentation” of CAP defined by a high risk of early clinical deterioration in order to target intensified management interventions to patients with a high potential of prognosis improvement ^47, 52^. A management-based risk stratification to allocate interventions like monitoring of organ functions, guideline-concordant management of severe sepsis, and early parenteral antimicrobial combination treatment has been suggested ^45, 53^. Obviously, patients presenting with immediate need for mechanical ventilation or vasopressor treatment are identified as medical emergencies. However, a recent analysis from the CAPNETZ cohort showed that prognosis is poorest in patients not presenting with these “major criteria” but deteriorating in the short course of the disease ^47^. Independent predictors for early deterioration were vital sign abnormalities at presentation, highlighting the need for careful clinical patient evaluation. Recent studies have shown that the American Thoracic Society/Infectious Diseases Society of America (ATS/IDSA) minor criteria, which represent parameters of acute organ dysfunction, improve high-risk prediction ^47, 54– 56^. Their accuracy to predict organ replacement therapy (optimal cutoff > two criteria) has been confirmed in a meta-analysis ^56^, and outcome improvement after management intensification guided by these criteria in the emergency room has been demonstrated in an interventional trial ^57^. Therefore, clinical high-risk identification should focus on acute respiratory or extra-pulmonary sepsis- or comorbidity-associated organ dysfunction, and careful clinical evaluation should be complemented by regular assessment of vital sign abnormalities and the minor criteria as shown in Table 2.

**Table 2.** High-risk identification in the emergency department (including assessment of the ATS/IDSA minor criteria).

Clinical assessment, supplemented by evaluation of the following criteria:
• Presence of acute respiratory failure ○ Respiratory rate of 30/min or more ○ Arterial oxygen partial pressure/fractional inspired oxygen (paO _2_/FiO _2_) of 250 or less ○ Oxygen saturation of less than 90% ○ Multi-lobar infiltrate
• Presence of acute extra-pulmonary organ dysfunction ○ Systolic blood pressure of less than 90 mm Hg/hypotension requiring aggressive fluid resuscitation ○ Elevated lactate ○ Temperature of less than 36°C ○ New-onset mental confusion ○ Acute renal failure/blood urea nitrogen of more than 20 mg/dL ○ Leucocytes of less than 4000/µL ○ Thrombocytes of less than 100,000/µL
• Presence of instable comorbidity ○ Especially acute cardiovascular complication

ATS/IDSA, American Thoracic Society/Infectious Diseases Society of America.

## Adjuvant treatment

Despite adequate antimicrobial therapy, approximately 10% of all patients with CAP will not survive their current pulmonary infection, and mortality rates for severe CAP levelled off at 20% to 30% for the last few decades, especially if treatment failure occurs ^58^. An overwhelming immunological response is assumed to be one of the key pathophysiological mechanisms behind the stagnating mortality rates. Keeping in mind the empty pipeline for new antimicrobial agents and the increasing threat of antibiotic resistance, adjuvant therapeutic strategies beyond just killing the causative microbes are urgently needed and are now the focus of research. Medicinal modulation or suppression of the immunologic host response (e.g. by the application of corticosteroids) might be a valid approach. This concept has been successfully implemented in the treatment of different infectious conditions such as bacterial meningitis and septic shock.

Following the first positive corticosteroid trial in CAP by Confalonieri *et al.* ^59^, conflicting results mainly from small clinical trials and meta-analysis questioned the potential benefit, and systematic reviews asked for larger randomised trials ^60– 62^. In 2015, two randomised controlled trials (RCTs) of adequate sample size were published, readopting the concept of adjuvant corticosteroid treatment in CAP ^63, 64^. Both studies appear to be supportive of corticosteroid administration, but before changing clinical practice, it is worth taking a closer look at their findings.

Blum *et al.* randomly assigned hospitalised patients with CAP (n = 785; 70% PSI III-V) to receive an adjuvant treatment with 50 mg prednisolone daily in addition to standard care ^63^. The primary endpoint of this study was time to clinical stability and it was reached 1.4 days earlier in the treatment group (3.0 versus 4.4 days, hazard ratio [HR] 1.33, *P* <0.001). Additionally, steroid administration facilitated an earlier switch to oral sequence therapy (4.0 versus 5.0 days, *P* = 0.011), shortened the length of hospitalisation (6.0 versus 7.0 days, HR 1.19, *P* = 0.012), and reduced the incidence of pneumonia-associated complications like acute respiratory distress syndrome (ARDS) and empyema (OR 0.46, 95% confidence interval [CI] 0.22 to 0.98, *P* = 0.05). These effects were independent of PSI score at admission, initial levels of CRP, and underlying comorbidities such as COPD. Unfortunately, the study was not designed to address mortality, the most important clinical outcome, and the number of side effects (hyperglycemia) was significantly higher in the treatment group (19% versus 11%, OR 1.96, *P* = 0.001). Furthermore, overall mortality was comparably low (3.4%) and only few patients required ICU treatment (4.8%).

The second study, by Torres *et al.*, included 120 CAP patients with high serum levels of inflammatory markers (CRP >150 mg/L) ^64^. The patients were randomly assigned to receive 0.5 mg/kg methylprednisolone given twice a day for 5 days or placebo. The primary endpoint was occurrence of treatment failure, a composite endpoint including early failure (progression to septic shock, the need for mechanical ventilation, or death within 72 hours after admission) and late failure (radiographic progression, persisting respiratory failure, progression to septic shock, the need for mechanical ventilation, or death within 72 to 120 hours after admission). The authors found significantly less treatment failure in the treatment group (13% versus 31%, *P* = 0.02) and by trend a lower mortality. Again, the incidence of hyperglycemia was higher, but not statistically significant (18% versus 12%, *P* = 0.34). Although the results were positive at first sight, a critical interpretation is recommended. The composite endpoint “treatment failure” allows for misinterpretations, especially when considering that the only significant differences between the groups were found in “late failure” due to “radiographic progression” and “late-onset septic shock”. “Radiographic progression” alone does not necessarily reflect clinical failure; it needs to be accompanied by clinical instability to be indicative of treatment failure, and “late-onset septic shock” is not always attributable to CAP.

In conclusion, there is growing evidence of some beneficial effects of steroid treatment in terms of faster resolution of clinical signs and symptoms and prevention of CAP-associated complications, but so far the impact on mortality cannot be judged sufficiently. This was confirmed by a recent meta-analysis indicating that steroids had no significant impact on mortality (relative risk [RR] 0.72, 95% CI 0.43 to 1.21), even in severe CAP (RR 0.72, 95% CI 0.43 to 1.21), but may prevent the development of ARDS (RR 0.21, 95% CI 0.08 to 0.59) and reduce the lengths of hospital and ICU stay and the time to clinical stability ^65^. Hopefully, the results of ongoing trials will help to elucidate the future role of adjuvant corticosteroid treatment in the management of CAP ^66, 67^. Nevertheless, it is important to notice that supportive steroid treatment in patients with influenza CAP is associated with increased mortality ^68^. Therefore, steroids should not be administered to patients with proven influenza, except for asthmatics and COPD patients who may require systemic steroids for the treatment of bronchial obstruction, even in the context of influenza ^69^.

Recently, it has been reported that statins may have immunomodulatory and anti-inflammatory properties and that their current intake may have favourable effects on the course of respiratory infections, including pneumonia ^70, 71^. However, most of the knowledge is derived from retrospective case-control studies including patients with already-established statin intake, and only few prospective RCTs are available.

In one large, population-based, case-control study (n >100,000), the recent use of statins significantly reduced the risk of mortality from pneumonia (adjusted OR 0.47) but had an effect neither on the incidence of non-severe pneumonia in the study population nor on the need for hospitalisation due to pneumonia ^72^. Two additional case-control studies found a 22% reduced risk of pneumonia in patients with current exposure to statins ^73, 74^. However, the protective effect was not shown in other studies, possibly indicating a “healthy user” bias ^75, 76^.

One recent randomised, double-blind, placebo-controlled trial addressed the effects of *de novo* statin use on CAP outcome and blood levels of inflammatory cytokines ^77^. In this study, the use of 20 mg simvastatin once daily for 4 days since hospital admission did not reduce the time to clinical stability and the levels of inflammatory cytokines in patients hospitalised with CAP. However, it is worth mentioning that the authors failed to achieve their recruitment target for determining the effect of *de novo* statin use on their clinical endpoint (time to clinical stability). Another randomised, double-blind, placebo-controlled trial focused on the effects of simvastatin (60 mg daily) on day 28 mortality in patients with ventilator-associated pneumonia ^78^. This study was prematurely stopped for futility after interim analyses with 300 patients enrolled. There was no difference in the primary endpoint (day 28 mortality) between groups (statin group 21.2% versus placebo group 15.2%, *P* = 0.1).

In conclusion, there is some evidence indicating that an established statin exposure may reduce the risk of pneumonia and have beneficial effects on the clinical course, but the results are conflicting and the findings of first RCTs on *de novo* statin use are discouraging. Therefore, the use of statins for primary prophylaxis or as adjuvant pneumonia therapy may not be recommended at present.

## Antibiotic treatment

Most major guidelines suggest an empiric treatment stratified according to severity of disease ^79, 80^. Outpatients are treated orally with penicillins, macrolides, tetracyclines, or fluoroquinolones with anti-pneumococcal activity (i.e. moxifloxacin or levofloxacin). Oral cephalosporins have been linked to increased treatment failure (OR 2.86, 95% CI 1.56 to 5.27), probably due to the unfavourable bioavailability plus—compared with intravenous administration—low licensed dosages ^81^. Recent EUCAST (European Committee on Antimicrobial Susceptibility Testing) guidelines state (e.g. for HI) that cefuroxime breakpoints apply only to high-dose treatment (i.e. 1.5 g three times daily).

As outlined above, the proportion of “atypical” pathogens has been probably overestimated in the past, particularly due to the low specificity of serology for detection of *Chlamydophila pneumoniae* ^82^.

Since coverage of atypical bacteria by macrolides, fluoroquinolones, or tetracyclines seems to be expandable in mild cases treated as outpatients—most guidelines recommend oral penicillins or aminopenicillins (longer half life time, higher bioavailability, and better activity against HI than penicillin V) to cover pneumococci—empiric combination treatment of inpatients with beta-lactam plus macrolide remains an issue of debate ^79, 80^.

Besides covering “atypical” pathogens, macrolides are supposed to attenuate the inflammatory response by decreasing expression of pro-inflammatory cytokines and consecutive neutrophil recruitment to lung parenchyma. A retrospective study revealed a clinical advantage of macrolides even in patients with macrolide-resistant pneumococcal pneumonia. However, recently, the cardiotoxicity of macrolides has been linked to a slightly increased mortality. A meta-analysis found that erythromycin carries the greatest risk of QT prolongation and torsades de pointes from all macrolides, followed by clarithromycin and azithromycin ^83^. A large Danish cohort study estimated 37 cardiac deaths in 1 million treatments with clarithromycin ^84^, with an increased risk particularly in women. Whereas the Svanstrom study addressed younger adults and not patients with CAP, the study by Ray *et al.* showed a higher risk of sudden death with azithromycin compared with amoxicillin ^85^. The study by Schembri *et al.* is currently the only one that looked specifically at protocol-defined CAP and showed an increased risk of long-term cardiac events ^86^. However, Mortensen *et al.* showed that the benefit of azithromycin in reducing CAP mortality outweighed the risk of cardiotoxicity ^87^.

A 2014 meta-analysis comprising four prospective cohort studies and 12 retrospective cohort studies (n = 42,942) found a decreased mortality for macrolide/beta-lactams versus beta-lactam monotherapy ^88^. However, randomised studies were not available.

Finally, in 2015, two randomised studies addressing this question were published. A cluster-randomised non-inferiority study from The Netherlands compared beta-lactam monotherapy, beta-lactam/macrolide, and fluoroquinolone for empiric treatment of CAP ^89^. A Swiss open-label, multi-centre, non-inferiority, randomised trial compared cefuroxime or amoxicillin/clavulanic acid with or without clarithromycin ^90^. The Swiss study could not prove non-inferiority for beta-lactam monotherapy regarding the proportion of patients reaching clinical stability on day 7, even after exclusion of patients with a positive urine legionella antigen test result. In contrast, the Dutch study found that beta-lactam monotherapy was non-inferior to strategies with a beta-lactam-macrolide combination or fluoroquinolone monotherapy with regard to 90-day mortality. The macrolide used in the Dutch study was erythromycin ^91^, which has a higher cardiotoxicity than azithromycin or clarithromycin ^83^. The Swiss study showed that, in particular, patients with atypical pathogens (mostly *Mycoplasma pneumoniae*) and patients with a higher severity profited from the macrolide combination. This supports the obligated empiric beta-lactam/macrolide combination treatment for at least all CAP patients admitted to the ICU, a strategy suggested by most major guidelines ^80, 81, 91^.

## Antibiotic stewardship

Antibiotic stewardship has become an important strategy to fight the antibiotic resistance crisis. How is antibiotic stewardship implemented in CAP treatment? First of all, pneumonia has to be differentiated from non-pneumonia entities (e.g. bronchitis and acute exacerbation of COPD) in patients presenting with lower respiratory tract infections. This requires a standard chest X-ray, which is frequently not available in the outpatient setting. Several studies have shown that using a PCT is a useful biomarker to decide for or against empiric antibiotics in inpatients and outpatients presenting with lower respiratory tract infections. PCT-guided strategies have decreased (unnecessary) antibiotic prescriptions by 30% to 50% without impairing clinical outcome ^92, 93^. Similar data are available in primary care for CRP, which—in contrast to PCT—is available as a point-of-care test ^94^.

Other approaches used a clinical score to predict CAP in outpatients presenting with acute respiratory tract infection in order to identify patients who should be prescribed antibiotics ^95^.

Another strategy to decrease antibiotic consumption without harming the patient is to shorten antibiotic treatment. A recent prospective before-and-after intervention study from Scotland describes the implementation of a simple CRB-65-based algorithm for duration of treatment (i.e. CAP: 5 days of antibiotics for mild and 7 for moderate/severe cases. Acute exacerbation of COPD: 5 days of antibiotics and no antibiotics at all in patients without an increase in sputum purulence) ^96^. This algorithm was enforced by automatic stop dates and pharmacist feedback to prescribers and resulted in significant reductions of antibiotic consumption and in antibiotic side effects without increasing mortality or length of stay.

### Vaccination

Vaccines are available against pneumococci and influenza virus, the most frequent bacterial and viral causes of CAP, respectively. Bacterial-viral co-infections are associated with increased mortality, and synergistic effects have been shown for combined vaccination ^12^.

The standard influenza vaccine is the trivalent split vaccine, containing two influenza A and one influenza B strains, which are annually selected by the World Health Organization. Within the last few years, efforts have been made to improve acceptance, coverage of the vaccine, and particularly its efficacy in the elderly. A central problem of the influenza vaccine is that the elderly, who are at increased risk, exhibit an inferior response to the vaccine because of immunosenescence. Intradermal vaccination aims to stimulate more antigen-presenting cells, which are found in higher concentration in the dermis than in the subcutis or the muscle. Virions mimic natural viral cell entry, and adjuvants aim to recruit more antigen-presenting cells. High-dose vaccines use four times the amount of antigen. Whereas studies have shown that most of these approaches increase antibody titres, only high-dose vaccines were tested in a study with a clinically relevant endpoint and showed an increased prevention of laboratory-confirmed influenza cases ^97^. Other strategies try to improve the influenza vaccine coverage. In contrast to influenza A, influenza B does not undergo antigenic shift and therefore does not cause pandemics. However, as a result of accumulated point mutations, influenza B split into two lines (Yamagata and Victoria) about 30 to 40 years ago ^98^. Historically, only one influenza B line was included in the trivalent split vaccine. Therefore, the coverage of the trivalent split vaccine has depended on the accurate prediction of the dominating B line in the particular season. Recently, quadrivalent influenza vaccines that include both influenza B lines have been made available for clinical use ^99^.

Pneumococcal vaccination of adults is a current issue of debate, since both a 23-valent polysaccharide vaccine and a 13-valent conjugate vaccine are licensed for use in adults. Meta-analyses have shown that the polysaccharide vaccine prevents pneumococcal bacteraemia with an efficacy of about 75%. However, the majority of pneumococcal pneumonia is non-bacteraemic, and it remains controversial whether this vaccine is protective against non-invasive pneumococcal pneumonia ^100^. To date, only one RCT in Japanese nursing home residents showed a clear reduction of pneumococcal pneumonia ^101^, whereas several other studies and a respective meta-analysis revealed no effect ^100^. In a recent meta-analysis comparing the four available RCTs on 23-valent polysaccharide pneumococcal vaccine (PPV23) efficacy against CAP, the study by Maruyama *et al.* ^101^ has been identified as an outlier, contributing statistically significant heterogeneity to this analysis ^102^. Recently, a large Dutch placebo-controlled multi-centre study showed that the 13-valent conjugate vaccine prevented non-invasive pneumococcal pneumonia due to vaccine serotypes with an efficacy of 45% ^103^. The coverage of 13-valent pneumococcal conjugate vaccine (PCV13) in adults is supposed to decrease because of herd protection effects of the PCV13 infant vaccination program that has led to a substantial decrease of the 13 vaccine serotypes in countries with such a program. Nevertheless, it remains unclear whether the herd protection effects on invasive pneumococcal disease seen after implementing PCV7 can be extrapolated to the additional six serotypes of PCV13 or non-invasive pneumococcal pneumonia or both. Recent data from Sweden, the US, and Germany suggest that there is only minor or no herd protection for serotype 3, one of the most frequent serotypes causing pneumonia in adults ^104– 106^. Considering these data, the Advisory Committee on Immunization Practices has suggested a sequential vaccination (PCV13 followed by PPV23 after 6 to 12 months) for all adults older than 65. Within the next few years, intensive surveillance on serotype distribution in pneumococcal pneumonia is needed in order to estimate the extension of herd protection and to evaluate the use of PCV13 vaccination in adults.

## Implications for clinical practice

CAP is the infectious disease with the highest number of deaths worldwide. Nevertheless, the importance of this disease is often underestimated. It is diagnosed too late, severity scoring is not appropriate, so that patients are too seldom admitted to intermediate care or ICUs, and antibiotic therapy is often not in accordance with guidelines. Large cohorts of patients with CAP have been established worldwide and vastly improved our knowledge about CAP. Therefore, current guidelines are much more evidence based than ever before. The challenge for the future is to implement current knowledge into clinical practice to reduce the number of CAP cases (by vaccination) and the number of deaths (by adequate diagnostics and treatment). National and international societies should establish CAP audits to oversee the management of CAP and to give clinicians feedback about their daily clinical practice.
